# Corrosion-Induced Stress Concentration Characteristics Study of Steel Strands in Bridge Cables Under Tension-Bending Coupling Loads

**DOI:** 10.3390/ma19040646

**Published:** 2026-02-07

**Authors:** Shaoling Ding, Xiyang Peng, Jian Xu, Dehao Ding, Huahuai Sun, Shunyao Cai

**Affiliations:** 1 School of Civil and Environmental Engineering, Changsha University of Science and Technology, Changsha 410114, China; dingshaoling@ccccltd.cn; 2 CCCC Second Highway Consultants Co., Ltd., Wuhan 430056, China; 3 School of Civil Engineering and Transportation, Yangzhou University, Yangzhou 225127, China; peng0316y@163.com; 4 Department of Construction Management, Chongqing University, Chongqing 400044, China; shunyao.cai@cqu.edu.cn

**Keywords:** steel strands, corrosion pits, pit morphology, tension and bending coupling loads, stress concentration

## Abstract

Steel strand cables, particularly in their anchorage zones, are simultaneously exposed to corrosive environments and subjected to tensile-bending coupling loads. Field observations of bridge cable failures indicate that they are primarily governed by the stress concentration characteristics of internally corroded steel strands under this complex stress state. Consequently, it is critical to investigate the corrosion-induced stress concentration characteristics of steel strands in bridge cables under realistic tension and bending coupling loads. An accelerated salt spray corrosion test was designed and conducted on steel strands in bridge cables. The mass loss rates of steel strands were analyzed to quantify varying degrees of corrosion. The morphological characteristics of corrosion pits on steel strands at different corrosion levels were observed and analyzed using a three-dimensional laser scanner. Based on the scanned data, the probability density functions for pit depth were fitted for each corrosion condition. Subsequently, a refined numerical methodology was developed to model corroded steel strands under tension-bending coupling loads. This methodology was utilized to perform a parametric study investigating the stress concentration characteristics at corrosion pits with different spatial dimensions of steel strands. The research results indicate that the mass loss rate of the steel strands increases nonlinearly with increasing corrosion duration. The depth of corrosion pits on the steel strands follows a Gaussian distribution across all investigated corrosion levels. The stress concentration factor of the corroded steel strands exhibits a significant linear correlation with the corrosion pit geometry. Specifically, the stress concentration factor decreases linearly with increasing corrosion pit length, but increases linearly with both corrosion pit width and depth. Quantitatively, a 0.1 mm increase in corrosion pit length, width, and depth results in decreases in the stress concentration factor by 0.062, increases by 0.036, and increases by 0.062, respectively.

## 1. Introduction

As the primary load-bearing components in cable-supported bridges, steel strand cables play a crucial role in transferring deck loads to the main cables or arch ribs, whose safety is directly linked to the normal operation and long-term durability of the whole bridge structure [1,2]. During service, the anchorage part of bridge cables is subjected to sustained tension-bending coupling loads [3,4], while corrosion is the most prevalent form of deterioration in the lower anchorage part of bridge cables [5]. Under the combined influence of corrosive environments and tension-bending coupling loads, cable failures frequently occur in the anchorage part, primarily manifesting as corrosion-fatigue fractures of steel wires, which has been validated through numerous case studies of cable failures in cable-supported bridges [6]. Such failure modes of bridge cables are predominantly governed by the stress concentration characteristics of internally corroded steel strands. Therefore, investigating the corrosion-induced stress concentration characteristics of steel strands in bridge cables under tension and bending coupling loads is of significant practical importance.

Given the extended timescale required for natural corrosion, accelerated corrosion tests are predominantly employed to investigate the morphological characteristics of corrosion pits, with initial research focus placed on high-strength steel wires. For instance, Nakamura et al. [7] conducted a one-year accelerated corrosion test on galvanized steel wires wrapped in gauze under wet-dry cycles. Results showed that most corrosion pits exhibited either circular or sharp triangular morphologies. Similarly, Li et al. [8] subjected high-strength steel wires to a three-month acetic acid salt spray test. According to the surface contours of corroded steel wires, they found that corrosion pit depth followed a Gumbel extreme value distribution. Further studies [9,10] have employed accelerated corrosion tests in neutral salt spray to achieve varying levels of corrosion on high-strength steel wires. From the corrosion morphology of corroded steel wires, four typical pit morphologies were identified, namely conical pit, hemispherical pit, combined pit, and secondary pit. The probability density functions of cross-sectional parameters for different corrosion degrees were modeled as normal distributions. More recently, Yan et al. [11] conducted a standardized salt spray accelerated corrosion test on steel wires and reconstructed the geometric features of corrosion pits on corroded steel wire surfaces. However, it should be noted that the aforementioned accelerated corrosion tests were conducted exclusively on stress-free steel wires, and thus did not account for the influence of the actual tension loads sustained by bridge cables. To address this limitation, researchers have begun to incorporate tensile stress into accelerated corrosion experiments. For instance, the statistical characteristics of corrosion morphology of steel wires were analyzed under varying strain levels and corrosion durations [12]. The established probability density functions for pit depth, radius, and center-to-center spacing indicated that pit depth followed an exponential distribution, whereas pit radius followed a lognormal distribution. Similarly, Fang et al. [13] investigated the geometric characteristics of surface pits on steel wires under tensile stress. They found that applied stress significantly influences pit propagation direction, leading to distinct pit morphologies under different stress conditions. In addition, electrochemical accelerated corrosion tests were conducted on steel wires to analyze the corrosion characteristics under different stress levels and corrosion durations [14]. The results found that the corrosion pit depth follows a lognormal distribution. Given the significant complexity of the actual service environment for bridge cables, the corrosion morphology observed in situ differs from that obtained under accelerated corrosion tests. Consequently, many researchers have directly examined the morphology of corroded steel wires extracted from decommissioned bridge cables. For example, Li et al. [15,16,17] investigated the characteristic parameters of pits on the surface of steel wires from suspenders of an arch bridge and the stay cables of a cable-stayed bridge. The pit characteristic parameters of steel wires conform to a lognormal probability distribution. Similarly, the distribution patterns of corrosion pits on steel wires extracted from an old arch bridge suspender were investigated [18]. Results indicated that the maximum pit depth was found to follow a Weibull distribution, while the maximum pit width and length conformed to a lognormal distribution. In summary, all the aforementioned studies have predominantly concentrated on characterizing the morphological features of corrosion pits on individual steel wires. However, as a prevalent type of bridge cable, steel strand cables possess a distinct helical geometric configuration. This inherent spiral structure, along with mechanical and electrochemical interactions among adjacent wires within the steel strand, may lead to fundamentally different corrosion morphologies compared to those observed in individual steel wires.

As research has progressed, the investigative focus has gradually expanded from individual steel wires to the more complex and representative case of steel strands. Vecchi et al. [19] examined the geometric morphology of corrosion pits on twenty-four steel strands extracted from 10-year-old, naturally corroded prestressed concrete beams. Complementing such field-based studies, Wang et al. [20] systematically investigated corrosion morphology under controlled laboratory conditions, subjecting steel strands to an artificial climate environment with specified stress levels, corrosion durations, and chloride ion concentrations. Compared to changes in surface morphology, the formation of corrosion pits is mechanically more critical, as they induce localized stress concentrations that serve as primary sites for fatigue crack initiation and growth, thereby constituting a key mechanism driving the degradation of the fatigue performance in bridge cables [21,22]. Building on this understanding, Yao et al. [23] employed finite element analysis to explore the nonlinear relationship between corrosion pit depth and the stress concentration factor in steel wires. Their results confirm that pit depth is the predominant factor influencing stress concentration. To probabilistically quantify this effect, Guo et al. [24] proposed a Bayesian prediction model for the stress concentration in corroded steel wires. Their Bayesian analysis revealed that the stress concentration factors around a corrosion pit are significantly influenced by the geometric aspect ratios of the pit, specifically its length-to-width and width-to-depth ratios. Subsequently, research has extended to consider the collective influence of multiple corrosion pits. For instance, Zhang et al. [25] systematically examined the impact of pit geometric parameters and spatial distribution patterns on the stress concentration factor of high-strength steel wires. Their findings revealed that the circumferential distribution of double pits exerts a more significant influence on the stress concentration factor compared to axial or oblique distributions, highlighting the critical role of relative pit orientation. Through integrated experimental and numerical analysis, Li et al. [26,27] investigated the influence of characteristic pit parameters on the stress concentration factor of corroded steel wires. They established a correlation between pit morphology and the stress concentration factor. However, the analytical focus of the studies cited above has been confined to individual high-strength steel wires. In contrast, the actual steel strands used in bridge cables possess a distinct helical geometry, typically characterized by a lay angle. This subtle yet critical structural difference fundamentally alters the characteristics of corrosion-induced stress concentration. Hence, Yu et al. [28] employed a multi-dimensional linear regression method to derive an empirical formula for predicting the stress concentration factor in steel strands. In addition, Fan et al. [29] studied the influence of pit characteristics on pit stress concentration, found that the depth of corrosion pits and the length–width ratio jointly affect stress concentration in steel strands, and established a dual-parameter stress concentration model for steel strands through numerical analysis. Most existing studies have focused exclusively on stress concentration in corroded steel strands under pure axial tension. In practice, however, bridge cable anchorage zones are subjected to tension-bending coupling loads, a condition under which stress concentration behavior fundamentally differs [30]. Since corrosion-induced stress concentration is a key driver of fatigue crack initiation, a quantitative link between three-dimensional pit geometry and the resulting stress concentration under realistic loading remains unestablished. Thus, a pronounced knowledge gap persists regarding corrosion-induced stress concentration under service-relevant tension-bending coupling loads based on actual corrosion pit morphology.

An accelerated salt spray corrosion test was designed and conducted on steel strands in bridge cables. The mass loss rates of steel strands were analyzed to quantify varying degrees of corrosion. A three-dimensional laser scanner was employed to observe and analyze the morphological characteristics of corrosion pits on strands at different corrosion levels. The probability density functions for pit depth were fitted and established for each corrosion condition. A refined numerical methodology was developed to model corroded steel strands under tension-bending coupling loads. Subsequently, this methodology was adopted to conduct a parametric study investigating the stress concentration characteristics at corrosion pits with different spatial dimensions of steel strands.

This study systematically elucidates the stress concentration in steel strands of bridge cables under tension-bending coupling loads, based on their actual corrosion pit morphology. By establishing a research perspective that more closely reflects the real-world combined action between the corrosive environment and tension-bending loads in anchorage zones, this work uncovers the underlying mechanical mechanisms that govern the failure mode of steel strands in bridge cable anchorage zones.

## 2. Salt Spray Accelerated Corrosion Test of Steel Strands

### 2.1. Steel Strand Specimens Treatment

**Figure 1:**
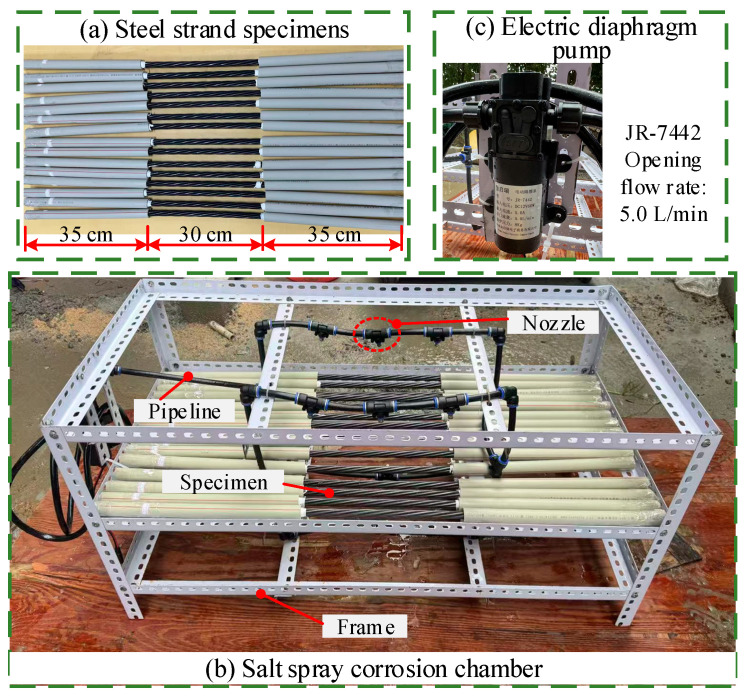
Salt spray accelerated corrosion test of steel strands: (**a**) steel strand specimens; (**b**) salt spray corrosion chamber; (**c**) the electric diaphragm pump.

The steel strand specimens used in the salt spray accelerated corrosion tests were galvanized strands (1 × 7-φ15.20) with a nominal diameter of 15.2 mm and a tensile strength of 1860 MPa. Each steel strand consisted of one straight central steel wire (diameter 5.20 mm) and six helical outer steel wires (diameter 5.00 mm), with a lay length of 220 mm. The density of steel strand specimens was 7850 kg/m^3^, the elastic modulus was 2.10 × 10^5^ MPa, and Poisson’s ratio was 0.30. A total of fifteen steel strand specimens, each 1.0 m in length and labeled from SN-1 to SN-15 (Figure 1a), were prepared. To achieve localized corrosion, only the central 30 cm section of each steel strand was exposed to the corrosive environment. The remaining portions were protected using a waterproof sealing procedure. The non-corrosion zones were first bound with waterproof cable ties, then sheathed with PVC tubing. The joints at both ends were sealed with adhesive tape to prevent the corrosive solution from entering. The prepared specimens are shown in Figure 1a. The initial mass of each treated specimen was measured using a JM-10002C electronic balance (precision of 0.01 g, Shanghai Liangping Instrument Co., Ltd., Shanghai, China) for subsequent corrosion mass loss calculation.

### 2.2. Fabrication of the Salt Spray Corrosion Chamber

The accelerated corrosion tests were conducted using a self-constructed salt spray chamber (Figure 1b). The chamber frame was constructed from triangular steel angles to ensure structural stability and load-bearing capacity during long-term testing. Its external dimensions were 1000 mm × 600 mm × 600 mm (Length × Width × Height), and it featured a three-layer design. The middle layer served as the placement zone for the steel strand specimens. The specimens were securely fixed at predetermined intervals, ensuring that the 30 cm long corrosion section of each specimen directly faced the salt spray nozzles. This configuration allowed the salt spray to act uniformly on the designated corrosion zones (Figure 1b for the specific arrangement). To provide a continuous and stable supply of salt spray, an electric diaphragm pump (JR-7442, opening flow rate: 5.0 L/min) was bolted to one side of the chamber (Figure 1c), ensuring reliable delivery of the corrosive solution to the nozzles. Eight nozzles were evenly distributed along the side walls, aligned with the corrosion sections of the specimens. The coordinated operation of these nozzles prevented localized unevenness in salt spray concentration.

During the test, the spray process was automatically controlled by an electric diaphragm pump coupled with a digital timer (KG316T), operating in an intermittent cycle to ensure precise timing. In accordance with the standard Corrosion tests in artificial atmospheres-Salt spray tests (GB/T 10125-2012) [31], each spray cycle lasted 2 h, with an active spray duration of 20 s per cycle. After installing and commissioning the pump, nozzles, and specimens inside the chamber, the entire unit was sealed with plastic film. This measure prevented the salt spray leakage and the resulting concentration attenuation, thereby maintaining a stable corrosion environment throughout the experiment.

### 2.3. Preparation of Corrosive Solution

To simulate the corrosive environment within bridge cable anchorage zones, this test employed a copper-accelerated acetic acid salt spray (CASS) solution. The solution was prepared using sodium chloride (NaCl), distilled water (H_2_O), cupric chloride dihydrate (CuCl_2_·2H_2_O), and glacial acetic acid (CH_3_COOH), with specific component proportions listed in Table 1. The preparation procedure was as follows. First, sodium chloride was dissolved in distilled water to obtain a base saline solution with a concentration of (50 ± 5) g/L. Next, cupric chloride dihydrate was added, with its concentration controlled within (0.26 ± 0.02) g/L. Finally, glacial acetic acid was introduced to adjust the acidity, maintaining a pH value between 3.0 and 3.3 at 25 °C. Prior to spraying, the solution was vacuum-filtered through an organic membrane to remove large particles that could clog the nozzles. The accelerated corrosion mechanism of the CASS solution is governed by micro-galvanic corrosion principles. The presence of Cu^2+^ ions allows for a displacement reaction where metallic copper deposits onto the steel surface. These copper deposits act as highly efficient cathodes, while the exposed steel matrix serves as the anode. Due to the significant potential difference between copper and iron, this coupling drives a high current density at the anodic sites, leading to rapid, localized pitting. Furthermore, the propagation of these pits is supported by the autocatalytic acidification theory. Inside the occluded pit, the hydrolysis of iron ions generates hydrogen ions, lowering the local pH. This acidic environment prevents the reformation of passive films and ensures the continuous vertical growth of the pit.

### 2.4. Test Conditions for Salt Spray Accelerated Corrosion

The salt spray accelerated corrosion test on steel strand specimens comprised five groups with different exposure durations (45, 75, 105, 135, and 165 days) and one uncorroded control group. For each target corrosion condition, accelerated corrosion tests were performed on unstressed specimens using a self-constructed salt spray chamber (Figure 1b). Owing to the chamber’s limited load capacity and size, it was not feasible to apply mechanical loading to the steel strand specimens simultaneously with corrosion exposure. Thus, all accelerated corrosion tests were conducted without applied stress. Although this setup simplifies real-world service conditions, it fulfills the primary research objective of characterizing corrosion pit morphology, thereby supplying realistic pit dimensions for the subsequent numerical modeling of corroded steel strands. The detailed parameters for each group are summarized in Table 2.

### 2.5. Mass Loss Analysis of Corroded Steel Strands

**Figure 2:**
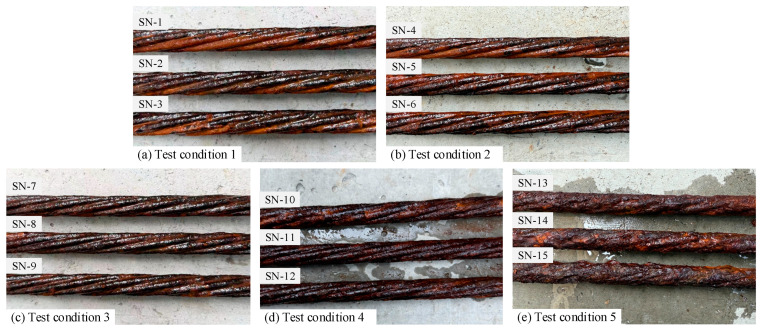
Morphology of steel strand specimens under different corrosion conditions: (**a**) test condition 1; (**b**) test condition 2; (**c**) test condition 3; (**d**) test condition 4; (**e**) test condition 5.

The surface morphology of the steel strand specimens under different corrosion conditions is shown in Figure 2. After exposure, the surfaces were covered with a dense, compact layer of reddish-black corrosion products. As the corrosion duration increased, the product color darkened and its coverage expanded across the steel wire surfaces. To reveal the underlying pit morphology, corrosion products were removed through an acid cleaning procedure consisting of pickling, rinsing, and drying. The specimens were immersed in diluted hydrochloric acid at room temperature for 24 h, then thoroughly rinsed. A soft brush was used to remove residual deposits. Finally, the specimens were dried in an electric blast oven at a constant temperature for 24 h to eliminate moisture from internal pores and surface residues. After cleaning, the steel strand surfaces regained a metallic luster, and corrosion pits of varying depths became clearly visible on the steel wire surfaces.

The mass of each corroded steel strand specimen was measured after the accelerated corrosion test. The mass loss proportion *η* of the corroded segment was adopted as a quantitative indicator to assess the degree of corrosion as follows:

(1) $$\eta =\frac{m_{0}-m_{1}}{m_{0}}\times 100\%$$

where *m*_0_ represents the initial mass of the steel strand specimen, and *m*_1_ denotes its mass after corrosion. The detailed mass-loss data for the steel strand specimens under each corrosion condition are summarized in Table 3.

**Figure 3:**
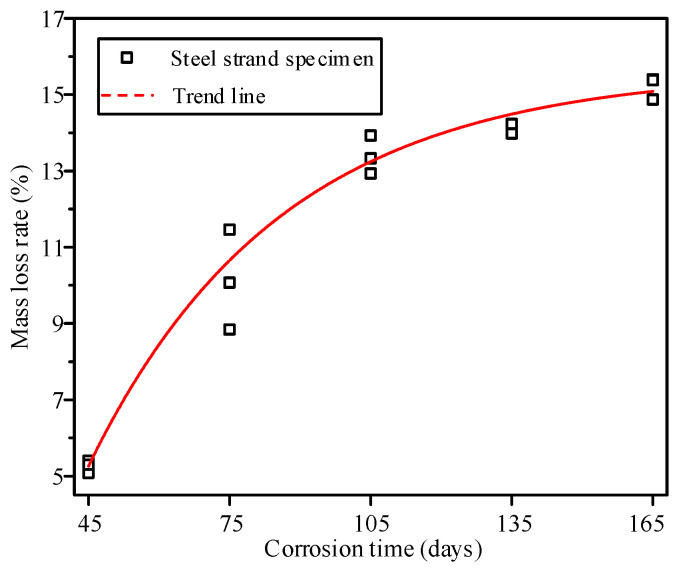
Mass loss proportion of corroded steel strands with different corrosion degrees.

The variation in the mass loss proportion of steel strands with corrosion duration is shown in Figure 3. The mass loss proportion increases nonlinearly with prolonged corrosion duration. In the initial stage, the mass loss proportion rises rapidly, but decelerates gradually in the later stage. This behavior reflects the stage-specific nature of the corrosion process. In the early phase, the absence of a continuous, dense corrosion product layer allows corrosive agents to directly access and erode the metal substrate, leading to a relatively high corrosion rate. As corrosion progresses, the accumulation of corrosion products in localized areas hinders the transport of corrosive agents toward the substrate interior, thereby slowing the rate. Furthermore, the dispersion of mass loss rates among specimens varies under different corrosion conditions. Greater dispersion is observed under conditions 2 and 3, while it is relatively smaller under other conditions. This variability originates from the inherent randomness of localized corrosion processes.

## 3. Morphological Characteristics of Corrosion Pits on Steel Strands

The width and length of corrosion pits exhibited significant irregularity, whereas their depth variation followed a clear and discernible trend. Therefore, the distribution patterns of pit depth were analyzed as the primary morphological indicator for steel strands at different corrosion levels. Pit depth measurements were obtained using a compound three-dimensional laser scanner.

### 3.1. Scanning of Corrosion Pit Depth

**Figure 4:**
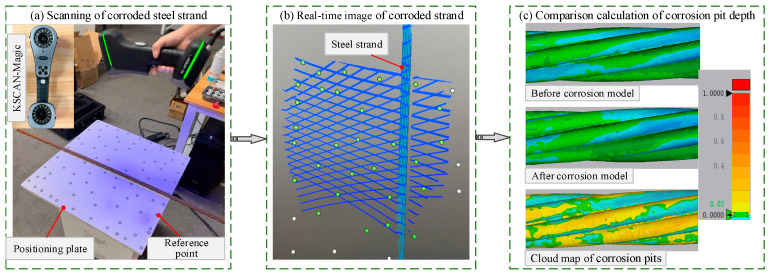
Scanning of corrosion pit morphology on steel strands: (**a**) scanning of corroded steel strand; (**b**) real-time image of corroded strand; (**c**) comparison calculation of corrosion pit depth.

A compound three-dimensional laser scanner (KSCAN-Magic) was employed to perform high-speed scanning of the corroded steel strand surfaces using 22 intersecting deep-blue laser lines (Figure 4a). The scanner offers a maximum accuracy of 0.020 mm and a maximum resolution of 0.010 mm. Given that the nominal diameter of the steel wire is 5.0 mm, this resolution provides a sufficient signal-to-noise ratio to accurately characterize the morphological characteristics of corrosion pits. The specimens were precisely positioned within marked areas on a positioning plate. The plate surface contained uniformly distributed reference points that served as spatial benchmarks for calibrating the observation areas during scanning. Throughout the process, real-time images (Figure 4b) were transmitted to a computer for subsequent analysis of corrosion pit depths.

The depth of corrosion pits was determined from the relative positional difference between the surfaces of the same steel strand before and after corrosion (Figure 4c). The original scan data for the uncorroded and corroded specimens were imported into Geomagic Wrap. Using the uncorroded model as the reference, the corroded model was aligned to it. Initial macro-scale positional deviations were corrected through automatic matching. The sampling frequencies of both models were then harmonized to ensure consistent point density and distribution. At this stage, the spatial alignment accurately represented the corrosion-induced surface morphology changes. The aligned models were subsequently imported into Control X software for surface deviation analysis. A three-dimensional spatial comparison was performed between the pre-corrosion and post-corrosion models to compute the height difference at each corresponding point. This procedure generated a surface deviation cloud map of the steel strand, as shown in Figure 4c.

### 3.2. Statistical Analysis of Corrosion Pit Depth

**Figure 5:**
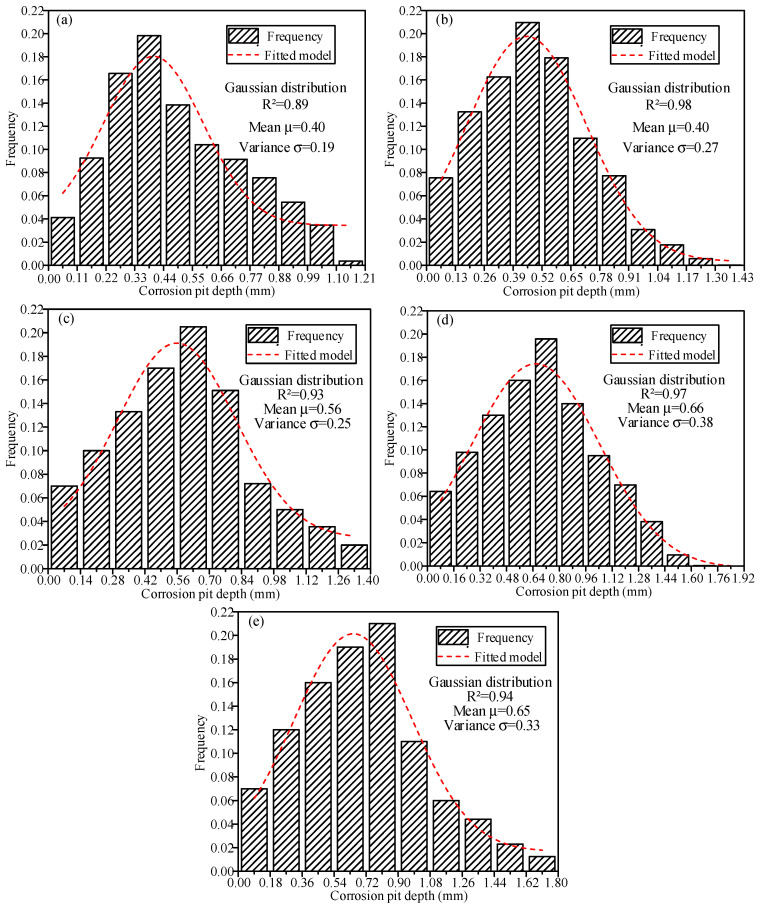
Histogram of frequency distribution of corrosion pit depth for steel strands under different corrosion conditions: (**a**) test condition 1; (**b**) test condition 2; (**c**) test condition 3; (**d**) test condition 4; (**e**) test condition 5.

Based on the pit depth data from all 15 specimens, a statistical analysis was performed for each corrosion condition. The corresponding frequency distribution histograms are presented in Figure 5. For condition C-1 (Figure 5a), the peak frequency of pit depth occurred in the interval of 0.33~0.44 mm. Beyond 0.44 mm, the frequency decreased gradually with depth, and the maximum pit depth exceeded 1.15 mm. Under condition C-2, the peak shifted to 0.39~0.52 mm, with frequency declining progressively beyond this range and a maximum depth of 1.23 mm. In condition C-3, the peak appeared near 0.7 mm; frequency decreased gradually beyond 0.77 mm, and the maximum depth reached 1.33 mm. For condition C-4, the highest frequency lay in 0.48~0.64 mm, beyond which frequency declined, and the maximum depth was 1.52 mm. Under condition C-5, the peak frequency was approximately 0.72 mm. Beyond 0.90 mm, frequency decreased rapidly, and the maximum depth increased to 1.71 mm. Across all conditions, the peak frequency remained around 20%. As shown in Figure 5, with prolonged corrosion duration, the distribution range of pit depths gradually widened, and the maximum pit depth increased. All conditions shared a common pattern: a high concentration of shallow pits, with frequency declining gradually with increasing depth. This pattern reflects the spatially cumulative and heterogeneous nature of corrosion in steel strands.

The frequency distribution of corrosion pit depths under each corrosion condition exhibited a unimodal pattern; therefore, this was fitted using a Gaussian distribution model (Figure 5) as follows:

(2) $$f(x)=\frac{1}{\sqrt{2\pi }\sigma }e^{-\frac{{\left(x-\mu \right)}^{2}}{2\sigma^{2}}}$$

where *μ* and *σ*^2^ are the mean and variance of the Gaussian distribution. The fitted parameters for each corrosion condition are summarized in Table 4. Except for condition C-1, all Gaussian models yielded R^2^ values above 0.90, indicating that the Gaussian distribution effectively captures the statistical characteristics of pit depth in steel strands under the tested conditions. The root mean square error (RMSE) of the fitted distribution under each condition is also listed in Table 4. RMSE reflects the overall deviation between the fitted model and the actual data, with lower values representing higher accuracy. As shown in Table 4, RMSE ranges from 5.59% to 7.00% across all conditions, demonstrating a high level of fitting accuracy for the pit depth distribution models. Furthermore, a cross-analysis between the mass loss data and the geometric data (3D scanning) confirms the internal consistency of the characterization techniques. As observed in Section 2.5, the mass loss rate decelerates in the later stages of corrosion (Conditions C-4 and C-5). This global trend is corroborated by the local 3D scanning data presented in Table 4, where the mean pit depth (*μ*) stabilizes at around 0.65–0.66 mm for Conditions C-4 and C-5. This convergence demonstrates that the high-precision laser scanning (0.02 mm accuracy) effectively captures the physical reality of the corrosion process. The synchronization between the reduction in mass loss rate and the stagnation of vertical pit growth reveals that corrosion product accumulation hinders further depth penetration.

To better understand the link between the corrosion conditions and the resulting pit structure, the mass loss behavior must be viewed through the lens of electrochemical kinetics. The observed nonlinear increase in mass loss (Figure 3) and the specific pit depth distributions (Figure 5) are governed by the interaction between the corrosive environment and the steel substrate. In the initial phase, the breakdown of the protective zinc layer and the passive film on the steel wire acts as the nucleation mechanism for pitting. As corrosion duration increases, the accumulation of corrosion products within the pits creates an occlusive effect. This leads to the formation of localized galvanic cells, with the pit bottom acting as a small anode and the surrounding surface as a large cathode. This electrochemical mechanism drives the vertical propagation of pits, resulting in the observed Gaussian distribution of pit depths.

## 4. Refined Numerical Method of Corroded Steel Strands Under Tension-Bending Coupling Loads

### 4.1. Numerical Modeling Method

**Figure 6:**
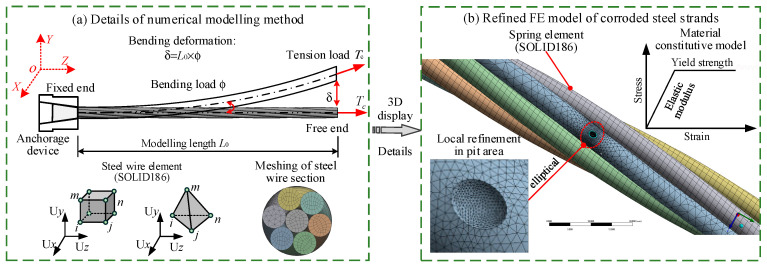
Refined numerical method of corroded steel strands under tension-bending coupling loads: (**a**) details of numerical modeling method; (**b**) refined FE model of corroded steel strands.

A refined three-dimensional solid model of a corroded 1 × 7-φ15.20 steel strand was developed in ANSYS Workbench (Version 2021 R1). The material was assumed to behave as an ideal elastoplastic, and no interfacial slip between the steel wires was permitted. The steel wires were modeled using SOLID186 elements (Figure 6a). Based on the observed corrosion morphology, corrosion pits were idealized as elliptical shapes (Figure 6b), as ellipsoidal pits represent the most prevalent morphology in corroded strands. Due to the geometric complexity introduced by the helical layout and the irregular pit geometry, purely hexahedral meshing was not feasible in pitted regions. Therefore, uncorroded steel wire sections were discretized with 1.0 mm hexahedral elements, while corroded sections, especially around pits, were meshed with tetrahedral elements, with local refinement applied in pit areas. Using the lay angle α and helical radius of each steel wire, the spatial configuration of the seven-wire strand was constructed, resulting in the structural model shown in Figure 6b. Boundary conditions were applied: fully fixed at one end and free at the other. All translational degrees of freedom (UX, UY, UZ) were constrained at the fixed-end nodes of the steel wire elements. At the free end, a tensile load *T*_c_ was applied to the solid element nodes of the steel wires along the global Z-direction (axial direction). To simulate bending induced by the rotation ϕ at the anchorage end, an equivalent bending displacement δ = *L*_0_ × ϕ was imposed on the solid element nodes of the steel wires at the free end. The refined finite element model of the corroded steel strand under tension-bending coupling loads was presented in Figure 6.

In the refined steel strand model, interactions between adjacent steel wires were simulated using frictional contact. Surface-to-surface contact pairs were automatically detected and defined in all potential contact zones based on the three-dimensional helical geometry of steel wires. The contact type was set as symmetric frictional contact with a coefficient of 0.115. The initial state assumed a tight fit between contact surfaces, with no initial gaps or preloads. The contact zones were defined based on the three-dimensional helical geometry of the steel wires, covering all surfaces where contact could potentially occur. To improve numerical stability and accuracy in friction simulation, the contact trimming function was activated with a trimming tolerance of 0.01 mm. The penalty function method was adopted to govern both normal and tangential behavior at the interfaces, with contact stiffness ranging from 0.1% to 10% of the steel wire elastic modulus. Interface settings were further refined to enhance geometric continuity with an offset of 0.01 mm specified to compensate for potential alignment deviations from modeling and meshing, and a no-ramp interface type was applied.

### 4.2. Numerical Analysis of Equivalent Stress at Corrosion Pits on Steel Strands

**Figure 7:**
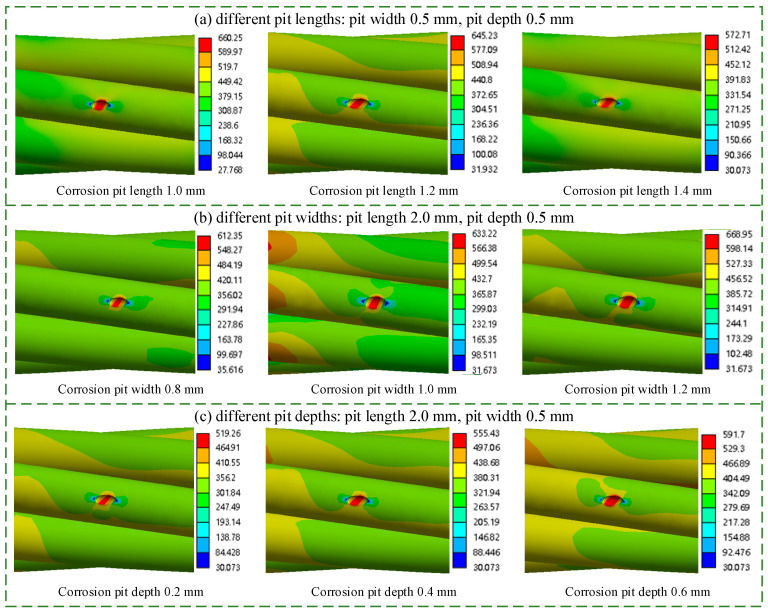
Equivalent stress at corrosion pits of different three-dimensional sizes on steel strands: (**a**) different pit lengths; (**b**) different pit widths; (**c**) different pit depths.

Based on the refined numerical model, the von Mises equivalent stress at corrosion pits was calculated under a tensile load *T*_c_ = 44.8 kN and a bending rotation ϕ = 0.6° at the anchorage end. The initial elliptical pit dimensions were set to 2.0 mm × 0.5 mm × 0.5 mm (length × width × depth), located at a longitudinal distance of 12 mm from the anchorage end. With pit width and depth fixed at 0.5 mm, the pit length was varied to 1.0 mm, 1.2 mm, and 1.4 mm in Figure 7a. The resulting maximum equivalent stresses were 660.3 MPa, 645.2 MPa, and 572.7 MPa, respectively, indicating a decreasing trend with increasing length. When the width of a single corrosion pit was set to 0.8 mm, 1.0 mm, and 1.2 mm, respectively, with the pit length fixed at 2.0 mm and the depth at 0.5 mm, the maximum equivalent stresses at the corrosion pit on the steel strand are shown in Figure 7b. Under these three width conditions, the maximum equivalent stresses at the corrosion pit were 612.4 MPa, 633.2 MPa, and 668.9 MPa, respectively. When the depth of a single corrosion pit was set to 0.2 mm, 0.4 mm, and 0.6 mm, respectively, with the pit length fixed at 2.0 mm and the width at 0.5 mm, the equivalent stress distribution at the corrosion pit on the steel strand is presented in Figure 7c. Under these three depth conditions, the maximum equivalent stresses at the corrosion pit progressively increased to 519.3 MPa, 555.4 MPa, and 591.7 MPa, respectively. These results demonstrate that significant stress concentration occurs at corrosion pits on steel strands, and that these characteristics are intrinsically governed by the three-dimensional morphology of the pits.

## 5. Parameter Studies of Stress Concentration Characteristics of Corroded Steel Strands Under Tension-Bending Coupling Loads

To demonstrate how the pit structure governs mechanical properties, the causal link between pit morphology and the stress concentration factor was analyzed. The presence of a corrosion pit disrupts the continuity of the material, forcing the internal force flow lines to deviate around the defect. The severity of this deviation and the resulting peak stress are strictly controlled by the geometric structure of the pit relative to the load direction. Under tension-bending coupling loads, the pit acts as a notch. The relationship is governed by notch sensitivity and the geometric transition gradient. A sharper pit structure induces a more abrupt change in the force transmission path, leading to a higher stress concentration factor as follows:

(3) $$K=\frac{\sigma_{\max}}{\sigma_{0}}$$

where *σ*_max_ is the maximum von Mises equivalent stress at the corrosion pit on the steel strand, and *σ*_0_ is the von Mises equivalent stress in the intact steel strand. Based on the refined numerical model described in Section 4, a parametric study was conducted to investigate the influence of three-dimensional pit morphology, specifically pit length, width, and depth, on the stress concentration characteristics of steel strands. The analysis was performed under a tension load *T*_c_ = 44.8 kN and a bending rotation ϕ = 0.6° at the anchorage end.

### 5.1. Effect of Corrosion Pit Length

**Figure 8:**
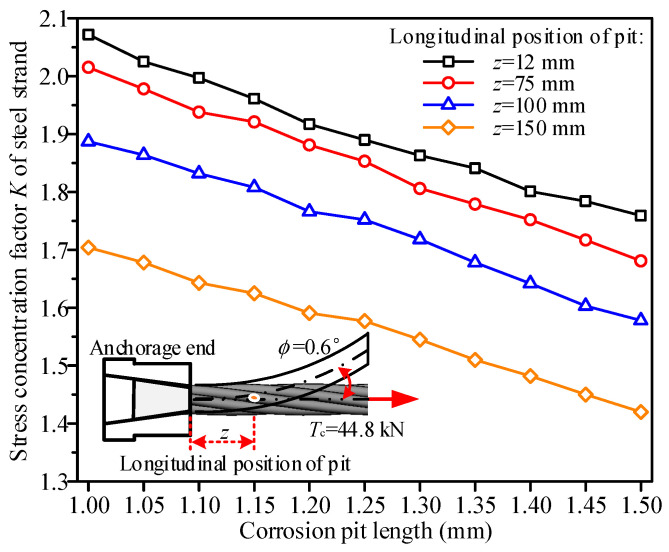
Effect of corrosion pit length on the stress concentration factor *K* of steel strands.

Under the condition of maintaining a pit width of 0.5 mm and depth of 0.5 mm, a numerical analysis was conducted to investigate the stress concentration characteristics of the steel strand under tension and bending coupling loads as the corrosion pit length increased from 1.00 mm to 1.50 mm, as shown in Figure 8. The results indicate that the effect of corrosion pit length on the stress concentration factor of the steel strand is significant and exhibits consistent trends across different longitudinal positions. For the corrosion pit at any longitudinal location, the stress concentration factor *K* of the steel strand decreases linearly with increasing corrosion pit length. When the corrosion pit length increased from 1.00 mm to 1.50 mm, the stress concentration factor *K* decreased from 2.07 to 1.76 for the corrosion pit located 12 mm from the anchorage end, from 2.02 to 1.68 for the corrosion pit at 75 mm, from 1.89 to 1.58 for the corrosion pit at 100 mm, and from 1.70 to 1.42 for the corrosion pit at 150 mm. The reduction in the stress concentration factor *K* across all longitudinal positions was approximately 16.2%. This phenomenon suggests that an increase in the corrosion pit length alleviates the stress concentration in steel strands under tension and bending coupling loads. The primary reason is that the steel wire surface becomes smoother as the corrosion pit length increases, thereby reducing the local geometric discontinuity on the steel wire surface. Quantitatively, for each 0.1 mm increase in corrosion pit length, the stress concentration factor decreases by 0.062. Additionally, as the longitudinal distance of the corrosion pit from the anchorage end increases, the stress concentration factor curve of the steel strand gradually shifts downward. Under identical pit length conditions, the closer the corrosion pit is to the anchorage end, the higher the stress concentration factor of the steel strand becomes. For example, at a corrosion pit length of 1.00 mm, the stress concentration factor was 2.07 for the corrosion pit located 12 mm from the anchorage end, whereas it was only 1.70 for the corrosion pit at 150 mm, representing a difference of approximately 15.4%. This is attributed to the tension and bending coupling loads acting on the steel strand, where the bending curvature induced by the anchorage-end rotation ϕ nonlinearly decreases along the longitudinal direction of the steel strand [3].

### 5.2. Effect of Corrosion Pit Width

**Figure 9:**
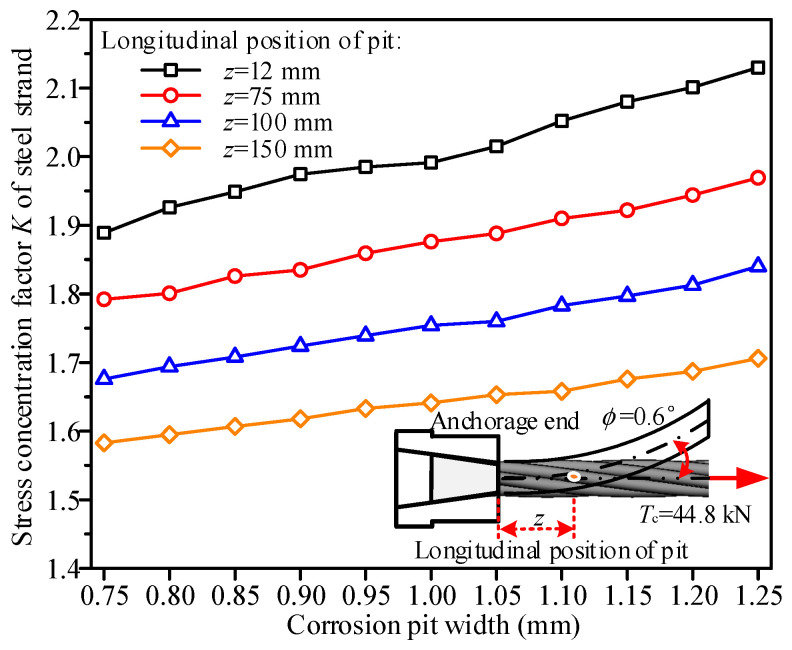
Effect of corrosion pit width on the stress concentration factor *K* of steel strands.

Under the condition of maintaining a pit length of 2.0 mm and a depth of 0.5 mm, a numerical analysis was conducted to investigate the stress concentration characteristics of the steel strand under tension and bending coupling loads as the pit width increased from 0.75 mm to 1.25 mm, as shown in Figure 9. The results indicate that the influence of corrosion pit width on the stress concentration factor of the steel strand follows a generally consistent trend across different longitudinal positions. Specifically, for pits at any longitudinal location, the stress concentration factor *K* of the steel strand increases linearly with increasing corrosion pit width. When the corrosion pit width increased from 0.75 mm to 1.25 mm, the stress concentration factor rose from 1.89 to 2.13 for the corrosion pit located 12 mm from the anchorage end, representing an increase of 12.7%. For the corrosion pit at 75 mm from the anchorage end, the factor *K* increased from 1.79 to 1.97 (10.0% increase). For the corrosion pit at 100 mm from the anchorage end, the factor *K* increased from 1.68 to 1.84 (9.5% increase). The factor *K* increased from 1.58 to 1.71 (8.2% increase) for the corrosion pit at 150 mm from the anchorage end. This phenomenon is likely attributed to the increased lateral width of the corrosion pit exacerbating the local geometric discontinuity of the helical circular steel wire surface, thereby intensifying stress concentration in the steel strand. Quantitatively, for each 0.1 mm increase in corrosion pit width, the stress concentration factor increases by 0.036. Furthermore, as the longitudinal distance of the corrosion pit from the anchorage end increases, the stress concentration factor curve gradually shifts downward, meaning that the closer the corrosion pit is to the anchorage end, the higher the stress concentration factor of the steel strand. For a corrosion pit width of 1.25 mm, the stress concentration factors were 2.13, 1.97, 1.84, and 1.71 for corrosion pits located 12 mm, 75 mm, 100 mm, and 150 mm from the anchorage end, respectively, resulting in a reduction of up to 19.7% in the stress concentration factor.

### 5.3. Effect of Corrosion Pit Depth

**Figure 10:**
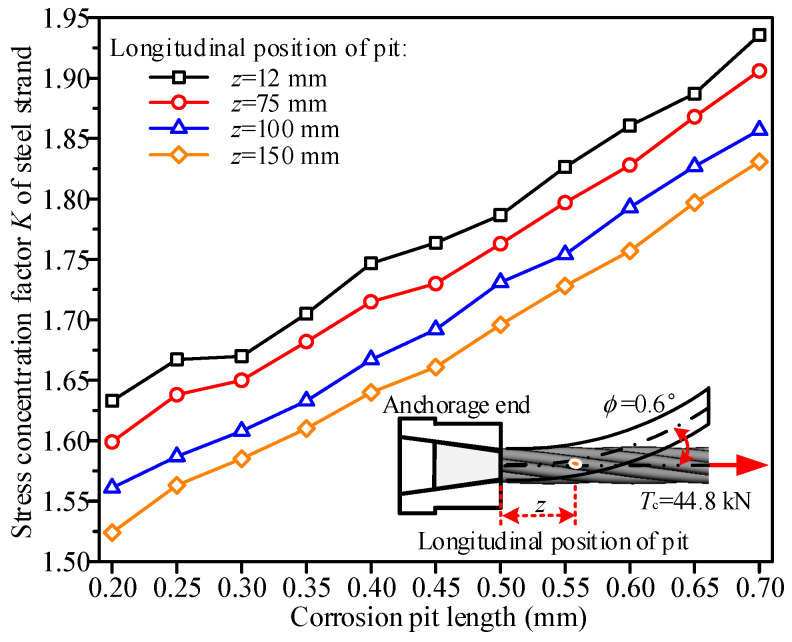
Effect of corrosion pit depth on the stress concentration factor *K* of steel strands.

Under the condition of maintaining a corrosion pit length of 2.0 mm and a width of 0.5 mm, a numerical analysis was conducted to investigate the stress concentration characteristics of the steel strand under tension and bending coupling loads as the pit depth increased from 0.20 mm to 0.70 mm, as shown in Figure 10. The results indicate that, for corrosion pits at different longitudinal positions, the variation pattern of the stress concentration factor *K* with the corrosion pit depth is generally consistent. Specifically, as the corrosion pit depth increases, the stress concentration factor of the steel strand exhibits an approximately linear growth. Slight deviations from linearity are observed at certain corrosion pit depths, which may be attributed to differences in local mesh discretization for corrosion pits of varying depths. When the corrosion pit depth increased from 0.20 mm to 0.70 mm, the stress concentration factor rose from 1.63 to 1.94 for the corrosion pit located 12 mm from the anchorage end, from 1.60 to 1.91 for the corrosion pit at 75 mm, from 1.56 to 1.86 for the corrosion pit at 100 mm, and from 1.52 to 1.83 for the corrosion pit at 150 mm. The increase in the stress concentration factor across all longitudinal positions was approximately 19.4%. This trend indicates that an increase in the corrosion pit depth exacerbates stress concentration in the steel strand, which can be attributed to the steeper geometric profile of the steel wire caused by deeper corrosion pits, leading to more pronounced local geometric discontinuities. Quantitatively, for each 0.1 mm increase in corrosion pit depth, the stress concentration factor increases by 0.062. Furthermore, for the same corrosion pit depth, the stress concentration factor is higher when the corrosion pit is closer to the anchorage end. Consequently, as the longitudinal distance of the corrosion pit from the anchorage end increases, the stress concentration factor curve gradually shifts downward. For a corrosion pit depth of 0.70 mm, the stress concentration factors were 1.94, 1.91, 1.86, and 1.83 for corrosion pits located 12 mm, 75 mm, 100 mm, and 150 mm from the anchorage end, respectively, resulting in a maximum difference of 6.0% in the stress concentration factor across different longitudinal positions.

## 6. Conclusions

An accelerated salt spray corrosion test was performed on steel strands, and the mass loss rates were quantified to evaluate different corrosion levels. The morphological characteristics of corrosion pits at varying corrosion degrees were observed and analyzed using a three-dimensional laser scanner. Subsequently, a refined numerical methodology was developed to simulate corroded steel strands under combined tension-bending loads and to investigate the associated stress concentration behavior at corrosion pits. Based on the integrated experimental and numerical results, the following conclusions can be drawn:

(1) The mass loss rate of the steel strands in bridge cables increases nonlinearly with prolonged corrosion duration. Specifically, it rises rapidly during the initial stage and then gradually decelerates in the later stage.

(2) Statistical analysis indicates that the depth of corrosion pits on the steel strands in bridge cables follows a Gaussian distribution across all investigated corrosion levels, with all fitted probability density models exhibiting residuals below 0.07, confirming their high accuracy.

(3) Under tension-bending coupling loads, the stress concentration factor of corroded steel strands in bridge cables exhibits a significant linear correlation with pit geometry. Specifically, the stress concentration factor decreases linearly with increasing pit length but increases linearly with increasing pit width and depth. Quantitatively, for each 0.1 mm increase in corrosion pit length, width, and depth, the stress concentration factor decreases by 0.062, increases by 0.036, and increases by 0.062, respectively.

This study investigated corrosion-induced stress concentration in steel strands under tension-bending coupling loads, offering novel insights into the mechanism of corrosion-fatigue degradation. The findings, specifically validated for high-strength bridge cable strands in an accelerated corrosive environment, establish a quantitative relationship between the stress concentration factor and three-dimensional pit dimensions. This relationship can be directly applied to the corrosion-fatigue assessment of bridge cables, thereby supporting their lifecycle management and maintenance.

Based on the known dimensions of corrosion pits, the numerical method developed in this study for corroded steel strands under tension-bending coupling loads is reproducible. Although the current analysis focuses solely on single-pit effects, the refined numerical method is highly scalable and can be used to investigate stress concentration in steel strands under complex multi-pit scenarios, which will also be explored in future work. A recognized limitation of this study is the qualitative nature of the numerical validation, due to the lack of direct experimental stress or strain data for correlation. To address this, future work will design tension- and bending-coupling loading tests for corroded steel strand specimens. Local strain at corrosion pits will be measured non-contact using Digital Image Correlation (DIC) technology under various loading conditions. These forthcoming experiments are expected to provide invaluable quantitative data for validating the presented numerical method under realistic service conditions.

## Figures and Tables

**Table 1:** Chemical composition of salt spray corrosive solution. Reprinted from Ref. [28].

Chemical Component	NaCl	H_2_O	CuCl_2_·2H_2_O	CH_3_COOH
Content per 5 L	250 g	4718.7 mL	1.3 g	30 mL
Mass percentage	5%	94.37%	0.03%	0.60%

**Table 2:** Test conditions for salt spray accelerated corrosion.

Test Conditions	Corrosion Durations (Days)	Number of Specimens	Corrosive Solution
C-0	0	3	5% NaCl solution with a mass fraction, and adjust its pH to 3.0~3.3 with acetic acid
C-1	45	3	
C-2	75	3	
C-3	105	3	
C-4	135	3	
C-5	165	3	

**Table 3:** Mass loss details of the steel strand specimens under different corrosion conditions.

Test Conditions	Specimen No.	Mass of the Corroded Part (g)		Mass Loss Proportion *η* (%)	
		Before Corrosion *m*_0_	After Corrosion *m*_1_	Individual Value	Average Value
C-1	SN-1	336.67	319.58	5.08	5.26%
	SN-2	334.66	316.59	5.40	
	SN-3	333.70	316.02	5.30	
C-2	SN-4	335.72	306.04	8.84	10.12%
	SN-5	337.32	298.67	11.46	
	SN-6	333.42	299.84	10.07	
C-3	SN-7	336.83	295.87	12.16	12.81%
	SN-8	336.55	293.04	12.93	
	SN-9	337.27	292.31	13.33	
C-4	SN-10	335.48	288.75	13.93	14.05%
	SN-11	336.07	288.26	14.23	
	SN-12	335.12	288.27	13.98	
C-5	SN-13	329.11	280.17	14.87	15.22%
	SN-14	330.79	279.85	15.40	
	SN-15	327.89	277.45	15.38	

**Table 4:** Details of the Gaussian model for the depth distribution of corrosion pits.

Test Conditions	Number of Corrosion Pits	Fitting Gauss Model Parameters			Maximum Frequency (%)	RMSE (%)
		Mean *μ*	Variance *σ*	R^2^		
C-1	1875	0.40	0.19	0.82	19.80	5.59
C-2	2052	0.40	0.27	0.98	20.95	7.00
C-3	2254	0.56	0.25	0.93	20.50	5.86
C-4	2452	0.66	0.38	0.97	19.58	6.16
C-5	2569	0.65	0.33	0.94	21.00	6.67

## Data Availability

The original contributions presented in this study are included in the article. Further inquiries can be directed to the corresponding author.
